# Assessing Genetic Overlap Between Platelet Parameters and Neurodegenerative Disorders

**DOI:** 10.3389/fimmu.2020.02127

**Published:** 2020-10-07

**Authors:** Alfonsina Tirozzi, Benedetta Izzi, Fabrizia Noro, Annalisa Marotta, Francesco Gianfagna, Marc F. Hoylaerts, Chiara Cerletti, Maria Benedetta Donati, Giovanni de Gaetano, Licia Iacoviello, Alessandro Gialluisi

**Affiliations:** ^1^Department of Epidemiology and Prevention, IRCCS NEUROMED, Pozzilli, Italy; ^2^Mediterranea Cardiocentro, Napoli, Italy; ^3^Department of Medicine and Surgery, University of Insubria, Varese, Italy; ^4^Department of Cardiovascular Sciences, Center for Molecular and Vascular Biology, University of Leuven, Leuven, Belgium

**Keywords:** neurodegenerative disorders, Parkinson disease, Alzheimer disease, platelets, genetics, platelet distribution width, genetic correlation

## Abstract

Neurodegenerative disorders such as Parkinson’s disease (PD) and Alzheimer’s disease (AD) suffer from the lack of risk-predictive circulating biomarkers, and clinical diagnosis occurs only when symptoms are evident. Among potential biomarkers, platelet parameters have been associated with both disorders. However, these associations have been scarcely investigated at the genetic level. Here, we tested genome-wide coheritability based on common genetic variants between platelet parameters and PD/AD risk, through Linkage Disequilibrium Score Regression. This revealed a significant genetic correlation between platelet distribution width (PDW), an index of platelet size variability, and PD risk (r_g_ [SE] = 0.080 [0.034]; p = 0.019), which was confirmed by a summary-summary polygenic score analysis, where PDW explained a small but significant proportion PD risk (<1%). AD risk showed no significant correlations, although a negative trend was observed with PDW (rg [SE] =-0.088 [0.053]; p=0.096), in line with previous epidemiological reports. These findings suggest the existence of limited shared genetic bases between PDW and PD and warrant further investigations to clarify the genes involved in this relation. Additionally, they suggest that the association between platelet parameters and AD risk is more environmental in nature, prompting an investigation into which factors may influence these traits.

## Introduction

Common neurodegenerative disorders due to accumulation of neurotoxic protein aggregates, such as Alzheimer’s disease (AD) and Parkinson’s disease (PD), suffer from the lack of risk-predictive circulating biomarkers, and clinical diagnosis occurs only when symptoms are evident, at an advanced stage of neurodegeneration (1, 2). Therefore, it is important to identify potential biomarkers that are easy to measure and that could predict the incident risk of such diseases, e.g., circulating biomarkers (3). Among these, platelets have received increasing attention (4–6), and their link with neurodegenerative disorders has long been hypothesized (7). Indeed, platelets are considered “circulating mirrors of neurons” and share many similarities with neural cells (8). These include the molecular machinery that controls the secretory system (5), patterns of reciprocal interactions, and the metabolism of different neurotransmitters like serotonin and dopamine, but also of neurologically important proteins like the Amyloid Precursor Protein (4).

In spite of these interesting cues, the relation between neurodegenerative disorders and classical blood platelet parameters like mean platelet volume (MPV), platelet count (Plt), and platelet distribution width (PDW) has been scarcely investigated at the epidemiological level, also with relatively common disorders like AD and PD. Observational studies consistently revealed an inverse association of PDW with AD and other forms of mild/severe dementia (9–11), and a positive association with cognitive performance (9, 11). An association of MPV with higher PD risk (12) was also reported but was not replicated in a later study, where MPV showed an increase with PD severity (13). At the genetic level, only two studies have previously investigated the relationship of blood platelet parameters with AD (14) and PD risk (15). In a large Genome Wide Association Scan (GWAS) testing associations of common genetic variants like Single Nucleotide Polymorphisms (SNPs) and small insertions/deletions (indels) with different blood cell measures (N_max_ ∼ 170,000) (14), the authors observed no evidence of a causal effect of Plt, MPV, or PDW on AD risk based on a multivariable Mendelian Randomization analysis. However, this technique may suffer from low power since it is usually based on a low number of variants (16). More recently, Nalls et al. (15) investigated genetic links of platelet parameters with PD risk through Linkage Disequilibrium (LD) Score Regression, which is a more robust approach based on hundreds of thousands of variants genome-wide (16) (see below). They reported non-significant genetic correlations with both Plt and MPV in the largest PD case-control GWAS meta-analysis carried out so far (involving ∼ 56,300 PD cases and ∼ 1.4 million controls) (15). Of note, in spite of the previous implication of PDW in neurodegenerative disorders (4, 9–11) and, more recently, in comorbid disorders like major depression (16, 17), this parameter has never been investigated with reference to PD risk at the genetic level.

Here, we tested the genetic relationship between the above mentioned platelet parameters, PD and AD risk, making use of summary statistics of large GWAS previously carried out on these traits (14, 15, 18). We first applied LD-score regression analysis to detect significant genome-wide co-heritability based on common genetic variants, and then we further investigated the significant correlations through polygenic risk association analysis (19). The aim of our investigation was twofold. First, we provided a comprehensive re-visitation of the genetic relationship between the most common neurodegenerative disorders—AD and PD—and platelet parameters commonly tested like Plt, MPV, and PDW, in a systematic and homogeneous way. Second, we provided hints into new potential biomarkers of such disorders, to drive future epidemiological, functional, and clinical studies.

## Methods

We applied LD-score regression (20, 21) to summary statistics of large independent GWAS previously conducted on AD (71,880 cases and 383,378 controls) (18), PD (54,376 cases and 1,474,097 controls), and platelet parameters, namely Plt, MPV, and PDW (N_max_ = 166,066) (14) (see **Table S1**). LD score regression models genetic correlation between two traits as a function of LD score among SNPs in 1 cM bins genome-wide, through the formula

$$r_{g}=\rho_{g}/\surd h_{1}^{2}*h_{2}^{2},$$

where ρ_g_ is the genetic covariance between trait 1 and trait 2, and $h_{1}^{2}$ and $h_{2}^{2}$ represent the SNP-based heritability of the two traits (20, 21). SNP-based heritability is in turn computed as the slope of the linear function between χ^2^ association statistics and LD score (i.e., the sum of r^2^ of a given SNP with all the other SNPs in a 1 cM window), for every SNP tested genome-wide (i.e., for which the association statistics are available in a given GWAS study). For this analysis, we filtered out variants that were not SNPs (e.g., indels), strand-ambiguous SNPs, and SNPs with duplicated rs numbers or Minor Allele Frequency (MAF) ≤ 1%. Moreover, SNPs with low values of sample size were also removed, when detailed information by SNP was available in the summary statistics file (N < 321,820 and < 301,340 for the PD and the AD GWAS, respectively). Finally, we retained only common SNPs (MAF > 5%) in the HapMap 3 EUR reference panel (22)—excluding the HLA region—since these variants have good imputation quality stats (r^2^>0.9) in most studies (21). LD scores of these variants were derived using the 1000G phase 1 v3 EUR panel (available at https://data.broadinstitute.org/alkesgroup/LDSCORE/w_hm3.snplist.bz2). Details on the number of variants available before and after quality control (QC) for each study are reported in **Table S1**.

Pairwise comparisons showing significant correlations were further investigated at a more fine-grained resolution, through a summary-summary polygenic risk score (Sum-Sum PRS) analysis using PRSice v1.25 (19). This method tests genetic overlap between two traits by making use of GWAS summary statistics: a training GWAS is used to build the PRS, which is then tested as a linear predictor of another trait in an independent study (target GWAS) (23). We performed Sum-Sum PRS analysis using only SNPs with association p-values (*P_T_*)  ≤ 0.05 in the training GWAS (on PDW) (14), in linkage equilibrium (r^2^ < 0.05) with the local top hit within a 300 kb window, and shared between the training (14) and the target GWAS (on PD risk) (15). To verify the robustness of our results, we repeated the analysis at increasing association significance thresholds in the training GWAS (with *P_T_* = 0.001, 0.05, 0.1, 0.2, 0.3, 0.4, 0.5, 0.6, 0.7, 0.8, 0.9, 1.0), as in (23). The number of SNPs meeting these criteria ranged from 2,813 (for *P_T_* ≤0.001) to 213,317 (for *P_T_* ≤1), respectively.

## Results and Discussion

**Figure 1:**
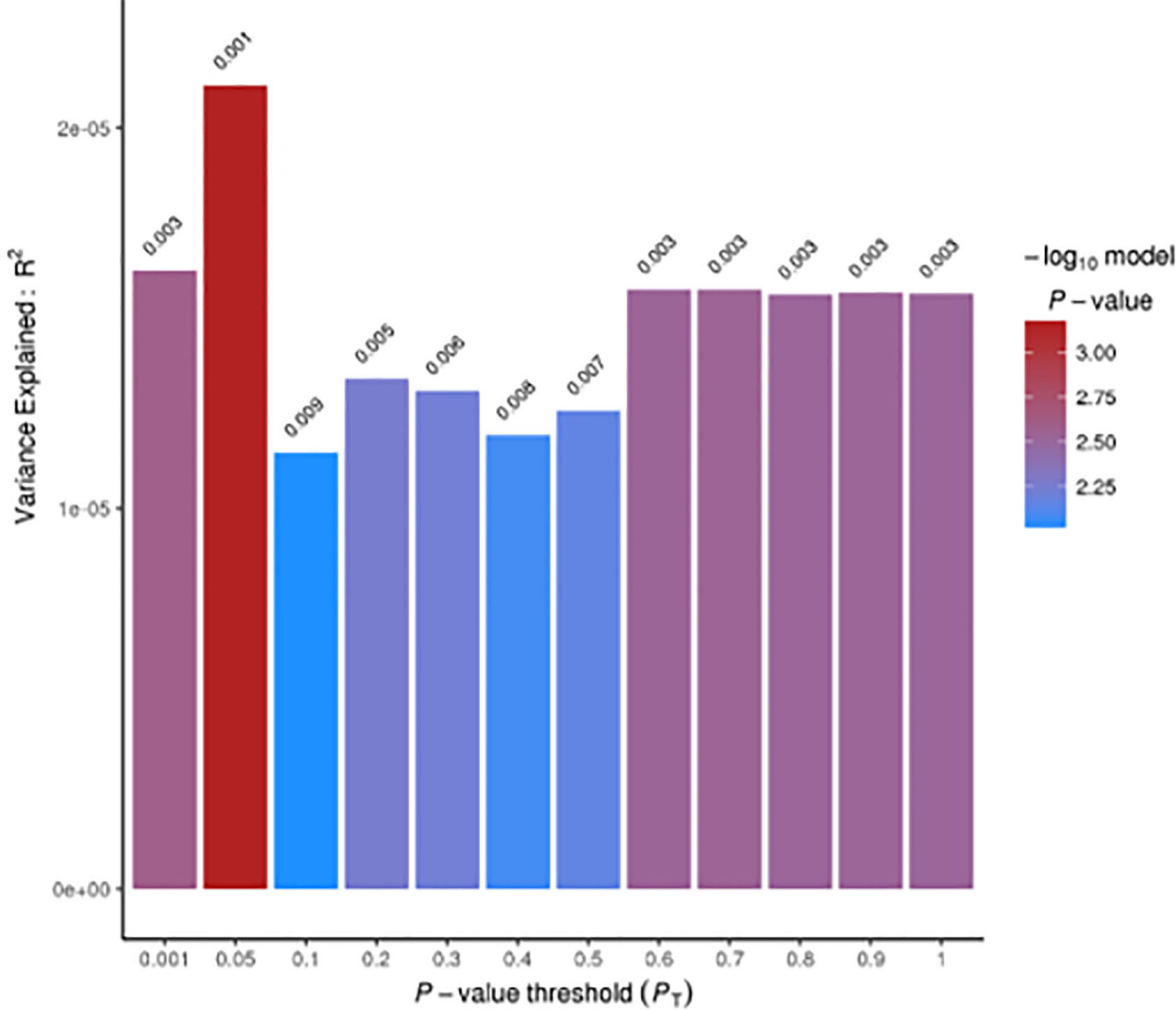
Summary-Summary polygenic risk score (Sum-Sum) analysis between PDW and Parkinson’s disease (PD) risk. No direction of effect could be inferred from Sum-Sum analysis, as per PRSice output (19).

LD score regression analysis revealed a significant genetic correlation between PDW and PD risk (r_g_ [Standard Error] = 0.080 [0.034]; p = 0.019; see **Tables 1A, B**) suggesting the existence of a genomic overlap based on common genetic variants. When we further analyzed this genetic relationship at a more fine-grained resolution, through Sum-Sum polygenic risk analysis, we observed that a modest but significant proportion of PD susceptibility (<1%) was explained by genetic variants nominally associated with PDW (at *P_T_* = 0.05: p = 7.0 × 10^−4^). This association was quite robust across varying p-value thresholds (*P_T_* ranging from 0.001 to 1.0; **Figure 1**). Overall, the evidence reported here suggests PDW as a new potential biomarker for Parkinson’s disease and is consistent with previous studies reporting positive associations between PDW and depression risk and/or symptoms, both at the epidemiological level (17, 24) and at the genetic level (16). Indeed, depression represents one of the main non-motor symptoms of PD, often presenting in its prodromal phase (25), and shows progressive patterns of microglial activation like other neurodegenerative disorders (26). Of note, previous epidemiological studies reported negative associations between PDW and the risk of cognitive impairment (9–11), which is co-morbid and partly shares biological bases with PD (27). While these two relations may appear in contrast, our knowledge of AD and PD is very limited from this point of view, and further epidemiological and clinical studies are needed to clarify the relation of PDW with the different neurodegenerative disorders, possibly through machine learning approaches including other platelet parameters and potential circulating biomarkers, to evaluate their prognostic value simultaneously. Similarly, genetic studies are warranted to identify specific genes influencing both PDW and PD risk.

**Table 1:** Genetic correlations of platelet parameters with **(A)** Parkinson’s disease and **(B)** Alzheimer’s disease risk, based on LD score regression analyses.

A					
Platelet parameter	#SNPs^a^	r_g_	SE	Z-score	p
Plt	916,946	-0.033	0.031	-1.07	0.28
MPV	916,936	0.046	0.031	1.47	0.14
PDW	**916,712**	**0.080**	**0.034**	**2.35**	**0.019**
**B**					
**Platelet parameter**	**#SNPs****^a^**	**r_g_**	**SE**	**Z-score**	**p**
Plt	1,123,504	0.016	0.052	0.30	0.77
MPV	1,123,487	0.010	0.051	0.19	0.85
PDW	1,123,214	-0.088	0.053	-1.66	0.096

Significant genetic correlations (p < 0.05) are highlighted in bold.

a Exact numbers of SNPs used to compute each pairwise genetic correlation (i.e., in common between the studies analyzed).

Plt, platelet count; MPV, mean platelet volume; PDW, platelet distribution width; SNPs, Single Nucleotide Polymorphisms; rg (SE), genetic correlation and relevant Standard Error.

By contrast, here we did not detect any significant genetic correlations between platelet parameters and AD risk (**Table 1B**), although PDW variability showed a trend of significance (rg [SE] = -0.088 [0.053]; p=0.096). This evidence suggests that the significant associations observed in previous epidemiological studies, which anyway showed a concordant sign (9–11), may be mainly due to shared environmental influences between platelet parameters and AD risk, and that common genetic influences are likely very limited, at least those of common variants. Indeed, a genetic link may have not been detected due to other (possibly rare or structural) genetic variants being at the basis of this. This hypothesis has been supported by recent findings for other complex traits like general cognition, educational attainment (28), and dyslexia (29), where only half of the heritability has been explained by common SNPs. Even so, this study rules out any large genetic overlap between PDW and AD risk.

In spite of the interesting findings reported here, the functional meaning of PDW and its potential usefulness as a biomarker remains to be clarified, beyond the neurodegenerative and neuropsychiatric landscape. As an index of heterogeneity of platelet size, reported associations of this marker with indices of platelet activation (30) suggest PDW might be a useful index of platelet function and procoagulant activity. This open issue, along with the modest co-heritability observed here, suggests caution in the interpretation of these findings and warrants further epidemiological, genetic, and functional studies to substantiate the potential usefulness of PDW as a new biomarker of neurodegeneration.

## Data Availability Statement

GWAS summary statistics analyzed in the present study are publicly available at the links reported in **Table S1**.

## Author Contributions

AG formulated the hypothesis, designed and performed statistical analyses. AT provided theoretical background and reviewed available literature. AG and AT wrote the manuscript, with contributions from all the co-authors. All the authors participated in discussion and interpretation of the results. All authors contributed to the article and approved the submitted version.

## Funding

AG and FN were supported by Fondazione Umberto Veronesi.

## Conflict of Interest

The authors declare that the research was conducted in the absence of any commercial or financial relationships that could be construed as a potential conflict of interest.
