# Supplementary material for: Assessing Genetic Overlap Between Platelet Parameters and Neurodegenerative Disorders
Source: Front Immunol. 2020 Oct 7;11:02127. doi: 10.3389/fimmu.2020.02127 (PMC7575686; doi:10.3389/fimmu.2020.02127)
Supplement: Supplementary file 1 [file DataSheet_1.pdf]

Supplementary Methods

Study	Phenotype	#variants before/after QC [a]	Ntot (Ncases/Nctrl) [b]	URL[c]
(1)	PD	10,674,335/922,182	1,528,473 (56,306/1,474,097)	https://drive.google.com/file/d/1FZ9UL99LAqyWnyNBxxlx6qOUlfAnublN/view?usp=sharing
(2)	AD	13,367,299/1,146,109	455,258 (71,880/383,378)	https://ctg.cncr.nl/software/summary_statistics
	Plt	29,522,061/1,180,459	166,066 (NA)	ftp://ftp.sanger.ac.uk/pub/project/humgen/summary_statistics/human/2017-12-12/
(3)	MPV	29,519,497/1,180,437	164,454 (NA)	ftp://ftp.sanger.ac.uk/pub/project/humgen/summary_statistics/human/2017-12-12/
	PDW	29,518,092/1,180,156	164,433 (NA)	

Table S1. GWAS studies which were used for the Linkage Disequilibrium (LD) score regression analysis.

[a] Number of genetic variants before and after quality control (QC) are reported for each study.

[b] Number of cases and controls are reported where applicable (AD and PD case-control GWAS studies).

[c] URLs where association statistics are available for download.

Abbreviations: AD = Alzheimer Disease; PD = Parkinson Disease; Plt = platelet count; MPV = mean platelet volume; PDW = platelet distribution width.

References

1. Nalls MA, Blauwendraat C, Vallerga CL, Heilbron K, Bandres-Ciga S, Chang D, Tan M, Kia DA, Noyce AJ, Xue A, et al. Expanding Parkinson's disease genetics: novel risk loci, genomic context, causal insights and heritable risk. *bioRxiv* (2019)388165. doi:10.1101/388165

2. Jansen IE, Savage JE, Watanabe K, Bryois J, Williams DM, Steinberg S, Sealock J, Karlsson IK, Hägg S, Athanasiu L, et al. Genome-wide meta-analysis identifies new loci and functional pathways influencing Alzheimer's disease risk. *Nat Genet* (2019) **51**:404–413. doi:10.1038/s41588-018-0311-9

3. Astle WJ, Elding H, Jiang T, Allen D, Ruklisa D, Mann AL, Mead D, Bouman H, Riveros-Mckay F, Kostadima MA, et al. The Allelic Landscape of Human Blood Cell Trait Variation and Links to Common Complex Disease. *Cell* (2016) **167**:1415-1429.e19. doi:10.1016/j.cell.2016.10.042
